# Challenges in Sequential Antiresorptive Therapy After Long-Term Denosumab Discontinuation: A Case Report and Narrative Review of the Literature

**DOI:** 10.3390/jcm15145443

**Published:** 2026-07-11

**Authors:** Maria-Evangelia Koloutsou, Melina Despina Pieper, Maria Mateniadou, Angeliki Papapanagiotou, Athanasios D. Anastasilakis, Polyzois Makras, Maria P. Yavropoulou

**Affiliations:** 1 Endocrinology Unit, 1st Department of Propaedeutic and Internal Medicine, School of Medicine, Laikon University Hospital of Athens, National and Kapodistrian University of Athens, 11527 Athens, Greece; mkoloutsou@med.uoa.gr (M.-E.K.); melinapieper2609@gmail.com (M.D.P.); smd2400288@uoa.gr (M.M.); myavropoulou@med.uoa.gr (M.P.Y.); 2 Department of Biological Chemistry, Medical School, National and Kapodistrian University of Athens, 15784 Athens, Greece; agpana@med.uoa.gr; 3 Department of Endocrinology, Diabetes and Metabolism, 424 General Military Hospital, 56429 Thessaloniki, Greece; a.anastasilakis@gmail.com; 4 Department of Endocrinology and Diabetes, 251 Hellenic Air Force & VA General Hospital, 3 Kanellopoulou St., 11525 Athens, Greece

**Keywords:** denosumab discontinuation, zoledronate, alendronate, osteoporosis, bone turnover markers, hepatotoxicity, inflammatory polyarthritis

## Abstract

**Background/Objectives:** Discontinuation of long-term denosumab (Dmab) remains a major clinical challenge because of rebound activation of bone remodeling and increased vertebral fracture risk. Intravenous zoledronate (ZOL) is widely recommended as sequential therapy, although evidence after very prolonged Dmab exposure is limited. We report a patient who developed two rare adverse events—acute hepatocellular injury and delayed inflammatory polyarthritis—following a single ZOL infusion administered after 12 years of continuous Dmab treatment. The subsequent management of persistent rebound bone turnover with oral alendronate (ALN) highlights the therapeutic challenges encountered when repeat ZOL administration is not feasible. **Methods:** A 65-year-old woman with postmenopausal osteoporosis received a single 5 mg ZOL infusion 6 months after her final Dmab injection following 12 years of continuous therapy. Within 24 h, she developed a typical acute phase response. Three days later, marked hepatocellular injury was detected, characterized by substantial elevations in transaminases and gamma-glutamyl transferase, while viral and autoimmune hepatitis were excluded. Liver enzymes normalized within five days with supportive management. Thirty-two days after ZOL administration, she developed inflammatory polyarthritis in the absence of previous rheumatologic disease and with negative immunologic testing. Treatment with low-dose prednisone (5 mg/day) resulted in rapid clinical and biochemical remission. At 6 months, bone turnover markers remained markedly elevated (CTX 0.82 ng/mL, P1NP 95 ng/mL), indicating insufficient suppression of rebound bone turnover after Dmab discontinuation. Because of the adverse events, the patient declined repeat ZOL administration. **Results:** Weekly oral alendronate (ALN) (70 mg) was initiated and was associated with partial suppression of bone turnover markers. Despite a decline in bone mineral density (BMD) of approximately 5% at both the lumbar spine and total hip over 12 months, BMD remained within the osteopenic range, and no new fragility fractures occurred during follow-up. **Conclusions:** This case illustrates two rare sequential immune-mediated adverse events following ZOL infusion and underscores the therapeutic challenges of managing Dmab discontinuation after long-term treatment when repeat ZOL administration is contraindicated. Sequential intravenous ZOL and oral ALN therapy was associated with partial suppression of bone turnover markers and protection from incident fractures during follow-up, despite a modest decline in BMD of approximately 5%.

## 1. Introduction

Zoledronate (ZOL), a potent intravenous aminobisphosphonate, is widely used in the management of osteoporosis and other skeletal disorders, including glucocorticoid-induced osteoporosis, Paget’s disease of bone, and cancer-associated bone disease [1].

In recent years, particular attention has been given to its role in mitigating the rebound increase in bone turnover and fracture risk observed after discontinuation of denosumab (Dmab), especially following long-term therapy [2].

Dmab discontinuation—or even delayed administration beyond the recommended 6-month interval—has been associated with a 3- to 5-fold increased risk of vertebral, major osteoporotic, and hip fractures [3]. Although the absolute proportion of affected patients appears relatively low (3.4–7.7% in prospectively studied cohorts), the clinical consequences can be severe, particularly in those treated for more than 2.5 years [4,5,6,7,8,9]. Administration of a single 5 mg infusion of ZOL 6 months after the last Dmab injection has been shown to preserve bone mineral density (BMD) gains in approximately 80% of patients previously treated with Dmab for about 2.5–3 years [10]. However, in patients exposed to longer durations of Dmab therapy (≥4–5 years), single ZOL may not fully prevent bone loss, and subsequent doses may be required [11].

Particular challenges arise in patients receiving Dmab for very prolonged periods. While long-term extension studies have demonstrated sustained efficacy and an acceptable safety profile for up to 10 years of treatment [12], evidence regarding optimal discontinuation strategies after more than a decade of therapy remains limited. Current ECTS recommendations suggest administration of ZOL approximately 6 months after the last Dmab injection, with subsequent monitoring of BTMs to guide the need for repeat antiresorptive treatment [2]. Prolonged suppression of bone remodeling may be associated with a more pronounced rebound increase in bone turnover following treatment cessation, and accumulating evidence suggests that a single ZOL infusion may provide less reliable protection against bone loss in patients exposed to Dmab for longer durations compared with those treated for only 2–3 years [13,14].

Consequently, management of Dmab discontinuation after very long-term treatment remains an important unresolved clinical challenge.

Emerging strategies suggest administration of ZOL at 6 months after the last Dmab injection followed by close monitoring of bone turnover markers (BTMs) at 3 and 6 months, with repeat antiresorptive treatment guided by biochemical response rather than annual BMD assessment [2].

Apart from ZOL, alendronate (ALN) and risedronate (RIS) have also been studied in this setting. Transition to ALN following short-term Dmab therapy appears to partially maintain BMD [15], whereas RIS has not shown sufficient efficacy in mitigating post-Dmab bone loss [16].

ZOL infusion is generally well tolerated. An acute phase response (APR), characterized by flu-like symptoms within 48 h, occurs in up to 50% of patients after the first infusion and decreases markedly with subsequent doses [17]. The underlying mechanism involves inhibition of farnesyl pyrophosphate synthase within the mevalonate pathway, leading to γδ T-cell activation and transient cytokine release [17]. Beyond APR, serious adverse events are uncommon and only sporadically reported [18].

We report a case of severe hepatotoxicity and subsequent inflammatory polyarthritis following the first ZOL infusion administered as part of a Dmab discontinuation strategy after 12 years of continuous treatment. Subsequent monitoring revealed a marked rebound increase in BTMs at 3 and 6 months, indicating reactivation of bone remodeling despite ZOL administration. Because repeat ZOL treatment was not feasible, oral ALN was initiated 6 months later and was associated with partial suppression of BTMs. Although BMD declined during follow-up, it remained within the osteopenic range, and no new fragility fractures occurred. This case highlights the challenges of managing Dmab discontinuation after very long-term therapy, underscores the potential for rare immune-mediated adverse reactions to ZOL, and suggests that oral antiresorptive therapy may represent a pragmatic alternative when further ZOL administration is contraindicated or declined. However, the contribution of ALN to the observed outcomes remains uncertain, and this approach should be considered hypothesis-generating.

### 1.1. Case Presentation

**Figure 1:**
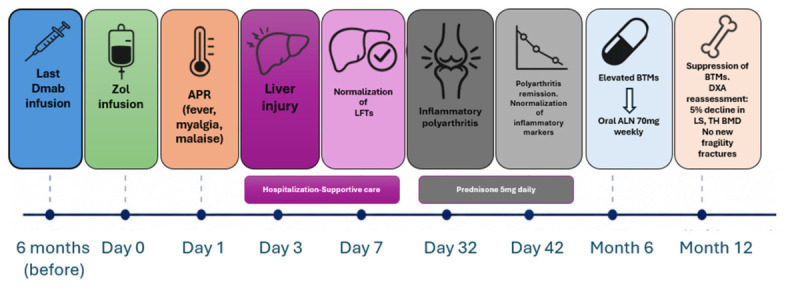
Chronological sequence of adverse events, treatment interventions, and skeletal outcomes following ZOL infusion. Abbreviations: Dmab, denosumab; ZOL, zoledronate; APR, acute phase response; LFTs, liver function tests; BTMs, bone turnover markers; ALN, alendronate; LS, lumbar spine; TH, total hip; BMD, bone mineral density, DXA, dual-energy X-ray absorptiometry. Different colors represent different categories of clinical events. Dashed vertical lines indicate the timing of each event along the study timeline.

A 65-year-old postmenopausal woman presented to the emergency department with profound fatigue of abrupt onset. She reported an intravenous infusion of ZOL (5 mg), administered three days earlier as sequential therapy for post-Dmab management that led to a typical APR within 24 h, characterized by high-grade fever up to 39.5 °C with chills, diffuse bone pain, and malaise, which resolved spontaneously on the second day (Figure 1).

**Figure 2:**
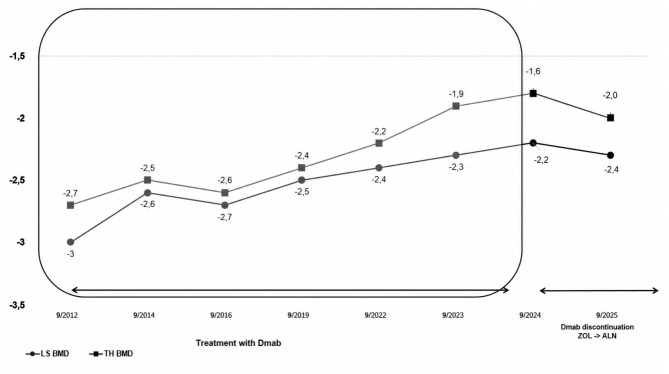
Changes in BMD expressed as T-scores, over 12 years of continuous Dmab therapy (60 mg subcutaneously every 6 months), followed by a single 5 mg intravenous infusion of ZOL administered 6 months after the final Dmab dose, and subsequent treatment with ALN (70 mg once weekly) initiated 6 months after ZOL infusion. BMD was measured by DXA (Hologic Inc., Marlborough, MA, USA). All DXA measurements were performed using the same Hologic densitometer (Hologic Inc., Marlborough, MA, USA) and APEX software version 5.6.1.1 throughout follow-up. Abbreviations: BMD, Bone Mineral Density; LS, Lumbar Spine; TH, Total Hip; Dmab, denosumab; ZOL, zoledronate; ALN, alendronate.

**Figure 3:**
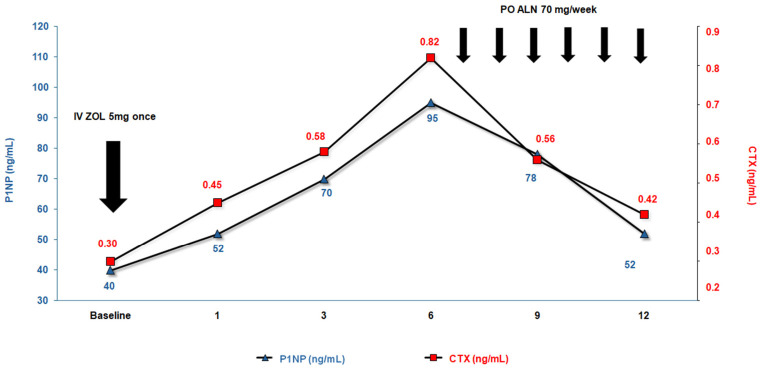
Changes in BTMs over 12 months following Dmab discontinuation and sequential antiresorptive treatment. Serum P1NP and CTX, were measured by electrochemiluminescence assay (ECLIA) on a Cobas e411 analyzer (Roche Diagnostics, Mannheim, Germany), with intra- and inter-assay coefficient of variation (CV) ≤ 4% and ≤3%, respectively, for P1NP and intra- and inter-assay coefficient of variation (CV) ≤ 3.5% and ≤8.4%, respectively, for CTX. Black arrows correspond to the months of ALN treatment. Abbreviations: BTMs, Bone Turnover Markers; Dmab, denosumab; ALN, alendronate; ZOL, zoledronate; P1NP, procollagen type I N-propeptide; CTX, C-terminal telopeptide of type I collagen, iv, intravenous; PO, per os.

The patient had postmenopausal osteoporosis with a right-sided Colles fragility fracture sustained 12 years earlier. She had been treated with Dmab for 12 consecutive years. Her BMD T-score at the end of the Dmab course was −2.2 (0.726 g/cm^2^) at the lumbar spine and −1.6 (0.803 g/cm^2^) at the total hip (Figure 2). Her baseline BTM values, namely procollagen type I N-propeptide (P1NP) and C-terminal telopeptide of type I collagen (CTX) were 40 ng/mL and 0.30 ng/mL, respectively (Figure 3).

Other comorbidities included (i) dyslipidemia, adequately controlled with atorvastatin 20 mg daily for several years without previous liver function abnormalities, (ii) hypothyroidism treated with levothyroxine 112 μg daily, and (iii) gastroesophageal reflux disease managed with intermittent proton pump inhibitor use.

On presentation to the emergency department, she appeared well-nourished and her vital signs were within normal values. Physical examination revealed no abnormalities, with no evidence of jaundice, rash, lymphadenopathy, or hepatosplenomegaly. Chest radiography and electrocardiography showed no acute pathology. Abdominal ultrasonography and Doppler evaluation of the splenoportal circulation was normal. Laboratory testing revealed significantly elevated transaminases and gamma-glutamyl transferase (GGT), while alkaline phosphatase (ALP), bilirubin, amylase, lipase, creatine kinase (CK), remained within normal limits (Table 1). Serologic testing for hepatotropic viruses, Human Immunodeficiency Virus and herpesviruses was negative, and autoimmune hepatitis was excluded based on the absence of immunologic markers (Table 1).

The patient denied recent use of acetaminophen, non-steroidal anti-inflammatory drugs, antibiotics, herbal preparations, or dietary supplements. She reported no recent vaccination, alcohol consumption, or symptoms suggestive of an intercurrent viral illness. Apart from long-standing atorvastatin and levothyroxine therapy and intermittent proton pump inhibitor use, there had been no recent medication initiation, discontinuation, or dose modification. Baseline liver function tests obtained immediately prior to ZOL administration were within the normal range (Table 1), providing a reference for the subsequent liver injury episode.

The differential diagnosis included acute viral hepatitis, autoimmune hepatitis, biliary obstruction, ischemic liver injury, and drug-induced liver injury. Serologic testing for hepatitis A, hepatitis B, hepatitis C, and HIV was negative, while autoimmune screening including ANA, AMA, SMA, and anti-LKM1 antibodies was also negative (Table 1). Abdominal ultrasonography and Doppler evaluation of the splenoportal circulation revealed no abnormalities. Testing for EBV, CMV, hepatitis E virus, and serum immunoglobulin levels was not performed. In the absence of an alternative explanation and given the close temporal relationship with ZOL administration, ZOL-associated hepatocellular injury was considered the most likely diagnosis.

She was hospitalized for intravenous hydration and close monitoring. Gradual biochemical improvement was reported, with liver enzyme levels decreasing by approximately 50% by day 2 and normalizing by day 5 (Figure 1).

Thirty-two days after the ZOL infusion, the patient presented again to our outpatient clinics with pronounced upper-limb arthralgia and joint pain (mainly in the radiocarpal and intercarpal joints), along with gait instability requiring a walker (Figure 1).

Clinical examination revealed inflammatory involvement of the wrists and small joints of the hands, characterized by tenderness, warmth, periarticular swelling, and prolonged stiffness with significant functional limitation.

To investigate the inflammatory polyarthritis, an extensive rheumatologic work-up was performed. Rheumatoid factor, anti-CCP antibodies, anti-dsDNA antibodies, anti-RNP antibodies, anti-Smith antibodies, anti-SSA/Ro and anti-SSB/La antibodies were all negative, while complement C3 and C4 levels were within the normal range, providing no evidence of an underlying autoimmune rheumatic disease (Table 1).

Low-dose prednisone (5 mg daily) was initiated, resulting in rapid clinical improvement with recovery of muscle strength and range of motion, accompanied by a decline in inflammatory markers, with erythrocyte sedimentation rate (ESR) decreasing from 83 mm/h (reference <20 mm/h) and C-reactive protein (CRP) from 163 mg/L (reference <5 mg/L) to within normal limits within 10 days of prednisolone treatment (Figure 1).

Musculoskeletal ultrasound, radiographic imaging, and joint aspiration were not performed because of the rapid clinical improvement following rheumatologic assessment and treatment initiation. Consequently, crystal-induced arthritis and other inflammatory arthritis could not be definitively excluded. However, given the absence of a previous rheumatologic history, the negative autoimmune evaluation, the temporal association with ZOL administration, and the prompt response to corticosteroid therapy, a ZOL-associated inflammatory arthritis was considered the most likely diagnosis.

At six months of follow-up, BTMs, P1NP and CTX were markedly elevated (CTX 0.82 ng/mL and P1NP 95 ng/mL), consistent with an ongoing overshooting of bone markers, insufficiently suppressed by the first ZOL infusion (Figure 1 and Figure 3). In line with the position statement by ECTS for Dmab discontinuation management [2], a second ZOL infusion was recommended. However, given the adverse events after the first infusion, the patient was reluctant to proceed. An individualized management approach was adopted, and oral ALN 70 mg weekly was initiated (Figure 1 and Figure 3). Subsequent follow-up demonstrated a progressive decline in BTMs while BMD remained within osteopenic range, with approximately 5% loss at LS and TH at 12 months (Figure 1 and Figure 2). Liver function tests and inflammatory markers remained within the normal range and no further adverse events or new fragility fractures were reported (Table 1).

### 1.2. Patient Perspective

The patient reported that the acute systemic reaction following ZOL infusion, the subsequent hospitalization for liver enzyme abnormalities, and the later development of inflammatory polyarthritis had a substantial impact on her quality of life and daily functioning. Although she was reassured by the gradual resolution of both adverse events, she remained highly concerned about the possibility of recurrence and therefore declined a second ZOL infusion despite understanding the potential risk of rebound bone loss after Dmab discontinuation. Following discussion of available alternatives, she agreed to initiate oral ALN and was satisfied with this treatment approach during follow-up, particularly because no further adverse events or fractures occurred.

## 2. Discussion

We report a case of two rare, sequential drug-induced adverse events—hepatotoxicity and inflammatory polyarthritis—occurring after an expected APR, in a patient who was administered a ZOL infusion as an exit strategy from long-term Dmab therapy. Delayed systemic adverse events, although uncommon, may be underrecognized, if prior ZOL exposure is not considered, underscoring the importance of clinical vigilance in patients receiving sequential antiresorptive therapy.

In the present case, a second ZOL infusion at 6 months would have been consistent with current recommendations in the presence of elevated BTMs, reflecting a rebound phenomenon after long-term Dmab therapy [2,3]. However, due to the severity of the adverse events following the first infusion, repeat ZOL administration was refused by the patient.

It should be emphasized that the clinically relevant aspect of this case is not the use of ALN per se, as oral ALN is an established antiresorptive option following Dmab discontinuation. Rather, the novelty lies in the combination of (i) exceptionally prolonged Dmab exposure (12 years), (ii) the inadequate suppression of rebound bone turnover following a single ZOL infusion, and (iii) the subsequent successful use of ALN when repeat ZOL administration was declined because of significant adverse events. Given the limited evidence available for oral bisphosphonate therapy after very long-term Dmab treatment, this case provides additional real-world experience that may help inform management of similar challenging clinical scenarios.

Current ECTS recommendations suggest that oral bisphosphonates may be considered following shorter duration of Dmab treatment, whereas intravenous ZOL once or twice in the first year is preferred after Dmab treatment exceeding 2.5 years [2]. Among the available oral BPs, RIS demonstrated inferior efficacy in preventing post-Dmab bone loss [16] while evidence supporting ALN after Dmab discontinuation is limited to patients with Dmab exposure of up to approximately 5 years [15,19,20,21].

Management of Dmab discontinuation has become an increasingly important clinical issue [22]. The increasing use of Dmab as a long-term therapy has resulted in a growing number of patients reaching treatment durations of 10 years or more. Long-term extension studies have demonstrated sustained efficacy and an acceptable safety profile over 10 years of continuous treatment [12,23,24]. However, evidence regarding the optimal management of Dmab discontinuation after more than 10 years of therapy remains scarce, and current recommendations are largely extrapolated from cohorts treated for substantially shorter periods [23,24].

ZOL has been widely adopted in this setting due to its high affinity for bone hydroxyapatite, potent antiresorptive effect, prolonged skeletal retention, and convenient annual administration, which promotes adherence [25]. The rationale for its use following Dmab discontinuation is primarily based on its greater potency and durability compared with oral bisphosphonates [22]. We have recently shown that in women receiving up to 22 denosumab injections (mean), 6 and 12 months of sequential ALN were similarly effective, associated with mean BMD losses of 5.9% at the LS and 3.5% at the FN observed over 12 months [26], comparable to the magnitude of bone loss reported after one or more ZOL infusions in patients discontinuing denosumab after approximately 4–5 years of treatment (range −4.0% to −5.5%) [5,14,27].

However, direct comparative studies between intravenous ZOL and oral ALN following long-term Dmab therapy are lacking [21]. Whether sequential or combination strategies provide additional benefit remains to be established.

Regarding safety, APR is the most common adverse event following ZOL infusion. In a large randomized controlled trial involving 7765 women, APR occurred in 42.4% of patients after the first ZOL infusion compared with 11.7% in the placebo group. Symptoms typically developed within 24 h, lasted a median of three days, and were mild to moderate in severity in approximately 90% of cases [18]. Consistent with these observations, our patient developed fever, malaise, myalgias, and arthralgias shortly after infusion, compatible with a typical APR [17,18,28]. However, the subsequent development of marked liver enzyme abnormalities and delayed inflammatory polyarthritis extended beyond the expected clinical spectrum of a conventional APR.

ZOL-associated hepatotoxicity has been described predominantly as an acute hepatocellular injury occurring within 72 h after infusion [29], typically preceded by an APR and resolving within 6–13 days under conservative management [18,29,30,31,32,33,34]. Published experience with ZOL-associated hepatotoxicity remains scarce, with only six cases reported to date [29] (Table 2). Most cases involved women receiving treatment for metabolic bone diseases, predominantly osteoporosis, and demonstrated a relatively consistent clinical course, with liver enzyme abnormalities developing within hours to 3 days after infusion typically preceded by a characteristic APR including fever, myalgias, arthralgias, or malaise (Table 2) [18]. The biochemical pattern was predominantly hepatocellular, and recovery was generally rapid and complete following supportive management (Table 2) [29]. Apart from a single reported case of autoimmune hepatitis requiring corticosteroids with prednisolone and immunosuppressive therapy with azathioprine, hepatotoxicity was self-limiting in all reported cases and resolved following supportive management (Table 2) [29]. In our patient, hepatotoxicity developed shortly after the APR and resolved rapidly with supportive care, consistent with previously described patterns (Table 2).

Inflammatory arthritis following ZOL infusion is also rare and has been reported mainly as flare-ups of pre-existing joint disease, usually occurring within days after infusion and resolving with conservative measures, including non-steroidal anti-inflammatory drugs or short-term corticosteroids (Table 3) [35,36]. Crystal-induced arthritis, including pseudogout, has also been described [37], whereas suppurative arthritis represents a distinct and rare entity (Table 3) [38]. Despite clinical heterogeneity, symptom onset generally occurs within hours to one week after infusion, and outcomes are favorable following supportive treatment, NSAIDs, corticosteroids, joint aspiration, or discontinuation of therapy (Table 3) [35,36,37,38,39,40,41,42]. Recurrence after re-exposure has also been documented, with a milder clinical course following subsequent infusions (Table 3) [35]. In contrast to most published cases, our patient had no prior history of rheumatologic disease and developed delayed-onset inflammatory polyarthritis approximately one month after infusion (Table 3). Rheumatologic evaluation favored a delayed ZOL-associated inflammatory arthritis, however, in the absence of imaging or synovial fluid analysis, crystal-induced arthritis and other inflammatory arthritis cannot be definitively excluded. The condition responded promptly to low-dose systemic corticosteroid therapy, suggesting an immune-mediated mechanism, however, delayed seronegative inflammatory arthritis unrelated to ZOL, although less likely, cannot be fully excluded.

To our knowledge, and based on a review of the published literature, we are not aware of previous report describing the sequential occurrence of both hepatotoxicity and delayed inflammatory polyarthritis following a single ZOL infusion administered as part of a Dmab discontinuation strategy. Although individual cases of ZOL-associated hepatotoxicity and inflammatory arthritis have been reported separately, the combination of both adverse events in the same patient appears to be exceedingly uncommon.

In the present case, sequential exposure to two bisphosphonates with different pharmacodynamic profiles—an initial high-potency intravenous infusion of ZOL followed by weekly oral ALN initiated 6 months later—was associated with partial suppression of the marked rebound increase in bone turnover markers at 6 months. BMD declined by approximately 5% at both the lumbar spine and total hip at 12 months, but remained within the osteopenic range and no new fragility fractures occurred during follow-up.

These findings suggest that, in patients with prolonged Dmab exposure, a single ZOL infusion may be insufficient to fully suppress rebound bone remodeling and preserve all prior BMD gains [13]. Although subsequent oral antiresorptive therapy may contribute to controlling the biochemical rebound, the extent to which it mitigates bone loss after very long-term Dmab treatment remains uncertain [23].

Several limitations should be acknowledged. First, this is a single case report, and a causal relationship between ZOL administration and the observed hepatotoxicity and inflammatory polyarthritis cannot be definitively established. Although the temporal association, exclusion of major alternative causes, and clinical course support a probable drug-related event, causality remains inferential. Second, rechallenge with ZOL was neither clinically appropriate nor acceptable to the patient because of the severity of the adverse events, precluding confirmation of causality. Third, because oral ALN was initiated after ZOL administration, the relative contribution of each agent to the subsequent partial suppression of bone turnover markers and preservation of bone mass cannot be determined. Furthermore, follow-up was limited, and the absence of new fragility fractures during the observation period should not be interpreted as evidence of fracture prevention. Finally, the management approach described here was individualized and should not be generalized to all patients discontinuing long-term Dmab therapy. Prospective studies are needed to better define optimal sequential treatment strategies in this challenging clinical setting.

In addition, although pharmacological therapy remains the cornerstone of osteoporosis management, long-term skeletal health also depends on a comprehensive approach that includes appropriate physical activity, muscle-strengthening exercise, fall-prevention strategies, adequate calcium and vitamin D intake, and regular reassessment of fracture risk [43]. Such measures may be particularly relevant in patients requiring individualized treatment plans following Dmab discontinuation [44,45].

Furthermore, when uncommon but clinically significant adverse events limit the use of standard treatment pathways, management must balance the risks of treatment-related complications against the potential consequences of uncontrolled bone loss and fracture [46]. Recent perspectives on the care of older adults emphasize that therapeutic decisions should be tailored to the individual patient, integrating disease severity, comorbidities, treatment tolerance, and patient preferences [47].

Overall, this case report incorporates the key elements recommended by contemporary case-reporting guidelines, including detailed documentation of the diagnostic evaluation, therapeutic interventions, clinical outcomes, follow-up, and patient perspective [48] and it highlights the clinical challenges of managing Dmab discontinuation after very prolonged therapy. The combination of a single ZOL infusion followed by oral ALN may represent an alternative in selected high-risk patients, however, its efficacy and safety remain hypothesis-generating, and prospective studies are needed to determine whether such an approach provides meaningful protection against bone loss and fractures in this setting.

## Figures and Tables

**Table 1:** Serial laboratory findings from baseline through 12 months of follow-up.

Parameter	Baseline (Pre-ZOL)	After-Zol				Reference Range
		Day 3	Day 32	6 Months	12 Months	
Creatinine (µmol/L)	55	53	58	56	57	45–84
AST (U/L)	22	**804**	24	21	23	<32
ALT (U/L)	19	**618**	26	22	24	<33
Alkaline phosphatase (U/L)	50	100	72	68	60	35–104
Gamma-glutamyl transferase (U/L)	21	**642**	28	24	26	5–36
Total bilirubin (µmol/L)	9.5	18.8	10.2	9.8	9.4	5.1–20.5
Direct bilirubin (µmol/L)	2.1	3.4	2.3	2.0	2.5	0–5.1
Albumin (g/L)	43	39	42	43	44	35–52
ESR (mm/h)	12	**85**	**83**	8	7	0–20
CRP (mg/L)	0.8	**47.1**	**163**	1.2	0.9	<5
Calcium (mmol/L)	2.3	2.2	2.3	2.4	2.3	2.1–2.5
Phosphate (mmol/L)	1.08	1.1	1.0	1.1	1.0	0.8–1.5
25(OH) Vitamin D (ng/mL)	32	31	30	34	35	20–50
PTH (pg/mL)	40	45	42	39	41	15–65
CTX (ng/mL)	0.30	–	–	0.82	0.42	0.17–1.00
P1NP (ng/mL)	40	–	–	95	52	20.2–76.3
HAV antibodies		Negative				
HBsAg		Negative				
Anti-HCV		Negative				
HIV		Negative				
ANA		Negative				
AMA		Negative				
SMA		Negative				
Anti-LKM1		Negative				
Anti-CCP antibodies (IU/mL)		0.7			<7	
Rheumatoid factor (IU/mL)		<10			<14	
Anti-dsDNA antibodies (IU/mL)		Negative				
Complement C3 (mg/dL)		134			90–230	
Complement C4 (mg/dL)		32			10–51	
Anti-RNP antibodies		<0.2			<1.0	
Anti-Smith antibodies		<0.2			<1.0	
Anti-SSA/Ro and Anti-SSB/La antibodies		Negative				

Abbreviations: SGOT, serumglutamic-oxaloacetictransaminase; SGPT, serum glutamic-pyruvictransaminase; ALP, alkaline phosphatase; PTH, parathyroid hormone; ESR: Erythrocyte Sedimentation Rate; CRP: C-Reactive Protein; CTX: C-terminal telopeptide of type I collagen; P1NP: Procollagen type I N-terminal propeptide; HAV antibodies, Hepatitis A virus antibodies; HBsAg, Hepatitis B surface antigen; Anti-HCV, Hepatitis C virus antibodies; HIV, Human Immunodeficiency Virus; ANA, Antinuclear Antibodies; AMA, Antimitochondrial Antibodies; SMA, Smooth Muscle Antibodies; Anti-LKM1, Anti-Liver Kidney Microsomal type 1 Antibodies; Anti-CCP, Anti-Cyclic Citrullinated Peptide Antibodies; Anti-dsDNA Antibodies, Anti-Double Stranded DNA Antibodies; Anti-RNP Antibodies, Anti-Ribonucleoprotein Antibodies; Anti-SSA/Ro Antibodies, Anti-Sjogren’s-syndrome-related Antigen A/Ro Antibodies; Anti-SSB/La Antibodies, Anti-Sjogren’s-syndrome-related Antigen B/La Antibodies. Bold values indicate abnormal laboratory values.

**Table 2:** Previous cases of ZOL-associated hepatotoxicity.

Author (Year)	Age/Sex	Indication	LFT Elevation/Normalization	Management
Polyzos et al. (2011) [30]	53/F	Paget disease	24 h/7 d	Supportive treatment
Lu Y et al. (2013) [31]	73/F	Primary osteoporosis	72 h/12 d	Hepatoprotective (polyene phosphatidylcholine) and supportive treatment
Jiang Y et al. (2015) [32]	50/F	Glucocorticoid-induced osteoporosis (Behçet disease)	72 h/9 d	Supportive treatment
Schneider et al. (2017) [34]	73/F	Primary osteoporosis	7 d/91 d	Corticosteroids (prednisolone) and immunosuppressive therapy (azathioprine)
Laway et al. (2022) [33]	55/F	Sheehan syndrome	24 h/6 d	Supportive treatment
Boldt et al. (2025) [29]	50/F	Aromatase inhibitor-induced osteoporosis (breast cancer)	12 h/13 d	Supportive treatment
Present case	65/F	Primary osteoporosis	72 h/5 d	Supportive treatment

Abbreviations: ZOL, zoledronate; LFT, liver function tests; F, female; h, hours; d, days. A targeted PubMed search using the terms ‘zoledronate’, ‘hepatotoxicity’, ‘drug-induced liver injury’, and ‘zoledronate acid adverse events’ was performed.

**Table 3:** Previous cases of ZOL-associated arthritis.

Author (Year)	Age/Sex	Indication/ZOL Exposure	Type of Arthritis	Time to Onset	Management	Outcome
Diaz-Borjon et al. (2006) [39]	51/F	Metastatic breast cancer/ After first ZOL infusion following 17 monthly pamidronate infusions, recurrence after pamidronate re-exposure	Recurrent bilateral knee arthritis with effusions	First hours	Discontinuation of bisphosphonate	Complete resolution, recurrence upon rechallenge
Werner de Castro et al. (2010) [35]	64/F	Osteoporosis/After first ZOL infusion, recurrence after second ZOL infusion	Inflammatory flare-up of hand osteoarthritis	First hours	Analgesia and intramuscular betamethasone	Complete recovery within 3 days, recurrence after second infusion with milder symptoms resolving within 2 days
Enomoto et al. (2012) [38]	70/M	Metastatic prostate cancer/After ~2 years of monthly ZOL infusions	Suppurative arthritis of the temporomandibular joint (TMJ)	After chronic exposure	Joint aspiration, IV antibiotics, surgical drainage, and prolonged irrigation	Marked clinical improvement, infection controlled, persistent fistula required ongoing local care
White et al. (2015) [40]	81/F	Osteoporosis/After first ZOL infusion	Acute polyarthritis	12 h	Hospitalization, analgesia, supportive treatment, physiotherapy	Gradual improvement, discharged home with intermediate-care support and physiotherapy
Hill et al. (2021) [37]	53/F	Osteoporosis/After first ZOL infusion	Acute pseudogout (CPPD arthritis)	1 day	NSAIDs and supportive care	Complete recovery
Florica et al. (2023) [41]	59/F	Osteoporosis/After first ZOL infusion	Acute polyarticular CPPD disease (pseudogout)	1 day	Corticosteroids and supportive care	Complete recovery
Wei et al. (2024) [42]	55/F	Osteoporosis/After first ZOL infusion	Bilateral knee arthritis with effusions	Within 1 week	Joint aspiration and corticosteroids	Progressive symptom resolution
Al Kiyumi et al. (2025) [36]	62/F	Osteoporosis/After first ZOL infusion	Flare-up of pre-existing osteoarthritis (knees, wrists, hands)	<24 h	Supportive treatment	Resolution within several days
Present case	65/F	Osteoporosis/After first ZOL infusion	Delayed inflammatory polyarthritis	32 days	Corticosteroids	Complete recovery

Abbreviations: ZOL, zoledronate; CPPD, calcium pyrophosphate deposition disease; TMJ, temporomandibular joint. A targeted PubMed search using the terms ‘zoledronate’, ‘arthritis’, ‘polyarthritis’, and ‘zoledronate adverse events’ was performed.

## Data Availability

The data supporting the findings of this study are available from the corresponding author upon reasonable request.
